# Right congenital diaphragmatic hernia associated with abnormality of the liver in adult

**DOI:** 10.11604/pamj.2017.28.70.11249

**Published:** 2017-09-22

**Authors:** Gezahen Negusse Ayane, Mikel Walsh, Jemal Shifa, Kadimo Khutsafalo

**Affiliations:** 1 University of Botswana, Faculty of Medicine, Botwana

**Keywords:** Bochdalek hernia (BH), hernia repair, congenital, colon, appendectomy

## Abstract

A Bochdalek hernia (BH) occurs when abdominal contents herniate through the postero-lateral segment of the diaphragm. The right side is affected considerably less commonly than the left. Most BHs present are diagnosed early in life, with some element of cardio-respiratory distress. Rarely, hernias that remain clinically silent until adulthood when they present as life-threatening surgical emergencies. We report a case 35 year old female who emergency exploratory laparotomy for a complete mechanical bowel obstruction. At surgery the redundant transverse colon was twisted and incarcerated within the right hemithorax, creating a closed loop obstruction. The right colon, appendix, terminal ilium, and three accessories right liver lobes were also dragged into the right thoracic cavity. After reducing the hernia, the diaphragmatic defect was primarily repaired with non-absorbable suture. The redundant transvers colon which had been compromised was resected and primary end-to- end anastomosis was carried out. Incidental appendectomy was done. The patient was sent into ICU for post-operative monitoring. She made an uneventful recovery and remains asymptomatic at nine month follow-up. I discuss what i believe to be the first case report of complicated right diaphragmatic hernia in Botswana, associated with another congenital mal-formation (accessories hepatic lobes, partial mal-rotation, and redundant transvers colon) in adult.

## Introduction

A diaphragmatic hernia may be congenital or secondary to traumatic injury of the diaphragm. The most common site of congenital or post-traumatic is into left side, congenital hernia develops trough the foramen of Bochdalek, which results from incomplete closure of the Pleuroperitoneal membrane in the postero-lateral portion of the diaphragm. The liver may also provide some measure of physical protection in to the right side. It is rare in adult and is most commonly reported on the left side of the diaphragm [1]. The size of the defects may be small enough to contain retroperitoneal fat or large enough to allow for the herniation of intra-abdominal organs. Therefore, BHs usually present with some element of respiratory distress following birth or in the early years of life. However, some patients remain asymptomatic until they are older. The diagnosis of BHs can be made during the prenatal period via the use of fetal ultrasonography (USG), but their late presentation in adults makes diagnosis more difficult. Hence, a careful physical examination combined with the use of imaging studies, such as multidetector computed tomography(MGCT), are needed to reach a correct diagnosis in adult patients suspected of having a BHs [1]. A Medline research showed only a few previous case reports of right-sided congenital Bochdalek hernia in adult. The most striking thought-provoking associated abnormality was the presence of multiple accessories right hepatic lobes.

## Patient and observation

**Figure 1:**
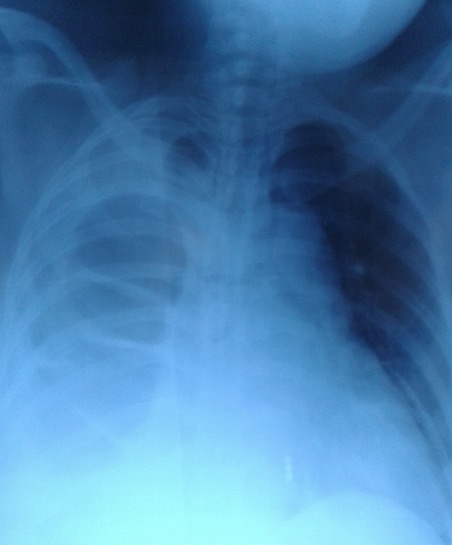
Chest X-ray showing gas filled bowel loops in the right hemithorax

A 35 year old female, presented with right sided chest pain, associated mild abdominal pain, and partial obstructive symptoms for several years. There was no significant past personal or family history). There was no history of trauma. Clinical examination revealed that she was mildly dehydrated, tachycardic (heart rate 110 beats per minute), afebrile, normotensive, and had mild abdominal tenderness. Investigation revealed a raised white cell count of 12.35x 10gl (reference range 4-10xgL). A chest radiograph revealed radio-opacity of right basal area (Figure 1) an abdominal erect radiograph was unremarkable. The patient was admitted with a presumed diagnosis of intra-abdominal tuberculosis, the medical officer was asked to do right thoracocentesis, which yielded a small amount of blood. While the patient was awaiting a chest and abdominal computed tomography (CT), she developed a mechanical intestinal obstruction. An emergency exploratory laparotomy was performed through a midline incision. We found a right sided Bochdalek's hernia containing transvers colon, right colon, along with the appendix and terminal ilium. The worrisome finding was that of a closed-loop intestinal obstruction. Incidentally, there were three accessory liver lobes, likely constituting the right lobe of the liver. The diaphragm had to be divided slightly to enable reduction of the incarcerated contents. These contents appeared compromised, but viable. Of particular interest, there was no hernia sac. The right lower lobe of the lung appeared hypoplastic. After reduction of the hernia contents, the diaphragmatic defect was repaired in a tension-free fashion. A limited resection and primary repair of the compromised redundant transvers colon was carried out. After careful consideration, we decided to carry out an incidental appendectomy. A right-sided chest tube was placed under direct vision, before closure of the diaphragmatic defect. The patient was transferred in to intensive care unit (ICU). She made an uneventful recovery and was discharged home. She was discharged home in excellent condition, and remains well at nine month follow-up (Figure 1).

## Discussion

The diaphragm is a large, dome-shaped structure composed of both muscle and tendon. It separates the pleural and peritoneal cavities, with the heart resting on the central tendon [2]. Embryologically, the diaphragm develops from four main structures. The septum transversum, arising from the mesoderm, forms an incomplete partition between the pericardial and peritoneal cavities. It will eventually become the central tendon of the diaphragm. The Pleuroperitoneal membranes fuse with the dorsal mesentery of the esophagus and the septum transversum to completely separate the thoracic and abdominal cavities. The dorsal mesentery of the esophagus eventually helps to form the diaphragmatic crura. As the lungs mature, the body-wall muscles are displaced and eventually contribute to the peripheral portions of the diaphragm [2]. Complete diaphragmatic formation takes place between the 4th and approximately 12 thweek of gestation [2]. Vincent Alexander Bochdalek described the fusion defect of the poster lateral foramina of the diaphragm in 1848 [3]. In the embryonic development period, fusion defects of the diaphragm can occur, resulting in postero-lateral defects (BH,95%), anterior-retrosternal defects (Morgagni,4%) and hiatal hernias and septum transversum defects (1%) [3]. The Morgagni Hernia (MH) is rare; it has been reported to occur in about 3% to 5% of all congenital diaphragmatic hernias (CDH) [4]. It is located to the left or right of the midline in the retrosternal position; the right is more common. Bochdalek hernia (BH) is the most common CDH, which occurs in the poster lateral portion of the diaphragm. The size of the hernia defect is variable and can range from 1cm to almost complete agenesis of the hemidiaphragm. Approximately 10% to 20% have a hernia sac at the site of a Bochdalek hernia. These hernias may contain stomach, spleen, colon omentum, and small bowel. The fact that the left poster lateral diaphragm closes after the right side may explain why the majority (~90%) of Bochdalek hernias occur on the left side. Right sided hernias are much rare and can involve the liver. Despite the high-speed, mechanized age in which we are living, traumatic right sided diaphragmatic hernias are extremely rare. This rarity with which we see traumatic right-sided diaphragmatic hernias may be explained by the fact that the liver shields the dome of the right diaphragm. It may also be that an injury of sufficient severity to produce such a hernia may often result in the death of such patients before surgical repair can be instituted [5]. In the congenital variety of diaphragmatic hernia, there may be associated malformations. These may be cardiac, gastrointestinal, or other. In newborns, the major problem accounting for morbidity and mortality is the associated pulmonary hypoplasia and hypertension [6]. In our case, the associated anomaly causing the greatest decision making challenge was the liver abnormality. Adhering to basic adult surgical principles allowed management of the hernia defect and compromised intestine (transverse colon). We questioned what, if anything, need be done regarding the abnormality of the right lobe of the liver. The correct decision seems to have been to reduce these accessory lobes, and not attempt to fix them. Indeed, to do so may have been meddlesome, and fraught with problems. We adopted a minimalist approach, repairing the obvious defect, resecting compromised bowel, and recognizing that while abnormal, the liver finding did not constitute a pathology warranting intervention, especially in the face of an incarcerated diaphragmatic hernia.

The clinical presentation of adult patients with right-sided Bochdalek hernia may vary from an incidental finding on radiological investigation to strangulation of contents with significant morbidity and mortality. Some report had documented patients with Bochdalek hernia suffering sudden death [3]. The initial diagnosis of a patient presenting with symptoms of CDH can be difficult and misleading. The recent widespread use of contemporary imaging tools in asymptomatic patients has yielded higher incidental rate. The treatment of BH is surgical and encompasses both reduction of the hernia contents and closure of the diaphragmatic defect. The hernia can be dealt with through thoracic or abdominal approaches and using minimally invasive or open technique, as well simple tension free suturing of the defect with non-absorbable sutures (herniorrhaphea) or mesh repair (hernioplasty). Polypropylene and expanded polytetrafluoroethylene (ePTFE) have been used good success. The open thoracic and abdominal approaches can be combined in difficult cases. Similar to the majority of literature reports, we chose the use an open abdominal approach for right Bochdalek hernia repair because: l): we did not have a clear diagnosis of BH pre-operatively 2): our patient presented with an abdominal emergency. Although the overall results of both thoracic and abdominal approaches are comparable, the approach of choice is a matter of personal preference and expertise, as there are no randomized studies providing the superiority of either method. The mortality for elective surgery has been reported to be less than 3%, but may be as high as 32% in an acute presentation with strangulation of herniated viscera.

## Conclusion

Adult right BH is a rare clinical entity. Its outcome depends on the timing of presentation, the early radiological confirmation of the diagnosis, and prompt intervention. Signs of peritonitis or intestinal obstruction mandate emergency intervention. Based on our experience, patients presenting with symptoms of intestinal and pulmonary conditions should be evaluated with BHs in mind.

## Competing interests

The author's declare no competing interests.
